# Evolution and Origin of HRS, a Protein Interacting with Merlin, the *Neurofibromatosis 2* Gene Product

**DOI:** 10.4137/grsb.s3106

**Published:** 2009-10-08

**Authors:** Leonid V. Omelyanchuk, Julia A. Pertseva, Sarah S. Burns, Long-Sheng Chang

**Affiliations:** 1 Institute of Cytology and Genetics, Russian Academy of Sciences, 10 Lavrent’ev Ave., 630090, Novosibirsk, Russia; 2 Center for Childhood Cancer, The Research Institute at Nationwide Children’s Hospital and Department of Pediatrics, The Ohio State University College of Medicine, 700 Children’s Drive, Columbus, OH 43205, USA

**Keywords:** hepatocyte growth factor receptor tyrosine kinase substrate, HRS, endosomal protein, Merlin

## Abstract

Hepatocyte growth factor receptor tyrosine kinase substrate (HRS) is an endosomal protein required for trafficking receptor tyrosine kinases from the early endosome to the lysosome. HRS interacts with Merlin, the *Neurofibromatosis 2* (*NF2*) gene product, and this interaction may be important for Merlin’s tumor suppressor activity. Understanding the evolution, origin, and structure of HRS may provide new insight into Merlin function. We show that HRS homologs are present across a wide range of Metazoa with the yeast Vps27 protein as their most distant ancestor. The phylogenetic tree of the HRS family coincides with species evolution and divergence, suggesting a unique function for HRS. Sequence alignment shows that various protein domains of HRS, including the VHS domain, the FYVE domain, the UIM domain, and the clathrin-binding domain, are conserved from yeast to multicellular organisms. The evolutionary transition from unicellular to multicellular organisms was accompanied by the appearance of a binding site for Merlin, which emerges in the early Metazoa after its separation from flatworms. In addition to the region responsible for growth suppression, the Merlin-binding and STAM-binding domains of HRS are conserved among multicellular organisms. The residue equivalent to tyrosine-377, which is phosphorylated in the human HRS protein, is highly conserved throughout the HRS family. Three additional conserved boxes lacking assigned functions are found in the HRS proteins of Metazoa. While boxes 1 and 3 may constitute the Eps-15-and Snx1-binding sites, respectively, box 2, containing the residue equivalent to tyrosine-377, is likely to be important for HRS phosphorylation. While several functional domains are conserved throughout the HRS family, the STAM-binding, Merlin-binding, and growth suppression domains evolved in the early Metazoa around the time the Merlin protein emerged. As these domains appear during the transition to multicellularity, their functional roles may be related to cell-cell interaction.

## Background

Bioinformatic and evolutionary studies that utilize genome sequencing data have advanced the molecular genetic analysis of genes and their functions in model organisms. The completed genomic sequences from a wide variety of species provide an opportunity to analyze the conserved protein domains and to define their origin in any given protein family. Furthermore, the presence of naturally-occurring sequence variations and alternatively spliced isoforms may help determine the details of protein function.

The activation of growth factor receptor tyrosine kinases, which leads to tyrosine phosphorylation of many cellular proteins, is a critical event in growth factor—mediated signaling transduction. Among the receptor tyrosine kinase substrates, the hepatocyte growth factor (HGF)—regulated tyrosine kinase substrate, abbreviated HRS, was first identified as a phosphotyrosine-containing protein, whose phosphorylation was rapidly induced in HGF-treated mouse melanoma cells.^1^ Cloning and sequencing analysis has revealed that HRS contains a zinc-finger domain, a proline-rich region, and a proline-and glutamine-rich region. Also, tyrosine phosphorylation of HRS was observed in cells treated with epidermal growth factor (EGF) or platelet-derived growth factor (PDGF). The human counterpart of HRS, termed HRS or HGS (OMIM *604375), was subsequently cloned as an interleukin-2 induced 110-kDa phosphotyrosine protein in MOLT-4 cells.^2^ HRS shares 93% amino acid sequence identity with that of the mouse HRS protein and contains double zinc finger (FYVE) motifs, a putative coiled-coil sequence, and nucleotide-binding sites. In addition, HRS interacts with STAM, a signal-transducing adaptor molecule that is directly associated with and phosphorylated by JAK3 and JAK2 upon stimulation with IL2 and GMCSF, respectively. The coiled-coil sequence appears to be important for the interaction between HRS and STAM.

HRS also shares 23% identical amino-acid sequence with Vps27, aproteinrequired for vesicular traffic in yeast. Using immunofluorescence and immunoelectron microscopy, Komada et al.^3^ showed that HRS localizes to the cytoplasmic surface of early endosomes. These results suggest that, like Vps27 in yeast, HRS may play an important role in vesicular transport via early endosomes in mammalian cells. Genetic knockout of HRS in mice has confirmed that HRS is involved in vesicular trafficking and required for ventral folding morphogenesis.^4^

In *Drosophila*, the HRS protein has also been implicated in endocytic regulation of receptor signaling, which is important in various developmental contexts.^5^ HRS facilitates the trafficking of ubiquitinated receptor tyrosine kinases (RTKs) to the degradation machinery, and *hrs* mutant cells fail to down-regulate the active RTKs, such as Torso and epidermal growth factor receptor (EGFR), leading to enhanced signaling.^6^–^8^ At the subcellular level, *hrs* mutants display enlarged endosomes caused by the inability of endosomes to invaginate their limited membrane to form multivesicular bodies.^4^,^6^ These results suggest that HRS-mediated receptor sorting into inner multivesicular body (MVB) vesicles is required for the attenuation of RTK signaling.

Several functional domains have been defined on HRS. The VHS domain, a domain commonly found in the Vps27, HRS, and STAM proteins, functions in cargo recognition in the trans-Golgi network.^9^ This domain has also been observed in proteins involved in signaling by growth factor receptors and receptor endocytosis, such as the EGFR-associated protein with SH3 and TAM (EAST).^10^ The FYVE domain, a zinc-finger-like domain first identified in the Fab1, YOTB/ZK632.12, Vac1, and EEA1 proteins, is responsible for the ability of HRS to bind to phosphatidylinositol 3-phosphate (PtdIns(3)P) in the cell membrane.^11^ The ubiquitin-interacting motif (UIM), originally described in the 26S proteasome subunit PSD4/RPN-10,^12^ is present in many proteins involved in endocytic events and vacuolar protein sorting.^13^ In addition to interacting with STAM,^2^,^14^ HRS binds to several other vesicular trafficking proteins, such as the synaptosome-associated protein SNAP-25, through the STAM-binding domain.^15^

HRS also interacts with Merlin, the product of the human *Neurofibromatosis 2* (*NF2*) gene, mutations in which cause neurofibromatosis type 2 (NF2).^16^–^19^ The HRS–Merlin interaction has been implicated in regulating Merlin’s tumor suppressor activity.^20^,^21^ Over-expression of HRS in rat schwannoma cells yields effects similar to those seen with Merlin over-expression, including growth inhibition, decreased motility, and abnormalities in cell spreading.^22^ Interaction with HRS may also help direct Merlin to its cortical localization.^15^ The observation that EGFR signaling is enhanced in *Merlin*-*expanded* double mutant clones due to reduced EGFR turnover^23^ suggests that Merlin’s intracellular localization is important for its ability to regulate RTK turnover.

By combining bioinformatic and phylogenetic approaches, we previously demonstrated a monophyletic origin of the Merlin protein in the early Metazoa.^24^ Amino acid sequence alignment of Merlin proteins from various species reveals several conserved protein binding domains. The conserved residues and structures identified correspond to the important sites highlighted by the available crystal structures for Merlin and the related Ezrin–Radixin–Moesin (ERM) proteins. The overall similarity between the primary and secondary structures of Merlin proteins from various species and the conservation of several functionally important residues suggest a universal role for Merlin in a wide range of Metazoa.

In contrast to the Merlin protein, the HRS homolog has been found in unicellular eukaryotes.^9^,^25^ Since Merlin and HRS physically interact with each other, it would be interesting to examine when the Merlin-interacting domain on HRS emerged during evolution. Also, examination of this and other interaction domains in various species may reveal conserved residues that are important for their interactions. To better understand the evolution, diversity, and overall distribution of HRS among different taxa, we performed bioinformatic analysis of HRS and examined the redundancy of the genes encoding HRS and the existence of its splicing variants.

## Results and Discussion

### BLAST identification of HRS sequences and assembly of predicted amino-acid sequences from whole genome shotgun

To identify putative HRS sequences in various eukaryotes, we performed a BLAST (database-uniprotkb, program-blastp, matrix-BLOSUM62, open gap penalty-11) search for homologous sequences in the database available at the ExPASy Proteomics Server (http://ca.expasy.org/) using the human HRS sequence (O14964) as a template. We cut off the protein homology search at an E-value of 4 e^−24^ (Vps protein of *Saccharomyces cerevisiae*), since the next homologous protein (*Coprinopsis cinerea*, A8NAI4) has an E-value of 1 e^−22^ producing an evident gap in an almost continuous series of E-values. The proteins, having only partial homology to O14964 or large deletions, were excluded from consideration to improve the quality of alignment.

The resulting protein sequences that showed homology extending over the entire length of the HRS protein were chosen for further analysis and included *Aedes aegypti* Q7PWQ2, *Bos taurus* BC111313 and Q0V8S0, *Mus musculus* Q3TLL4 (or Q3UMA3) and Q99LI8, *Rattus norvegicus* Q9JJ50, *Xenopus tropicalis* Q28CS1, *Ciona intestinalis* Q1RQ15, *Brachydanio rerio* Q6PH00, *Caenorhabditis elegans* Q17796, *Schistosoma japonicum* Q5C033, and *Homo sapiens* O14964 (Table 1). We identified several proteins that had not been previously annotated as HRS or HGS, including three unnamed sequences, Q29KX2 of *Drosophila pseudoobscura*, Q7PWQ2 of *Anopheles gambiae*, and Q4SE24 of *Tetraodon nigroviridis*, and two hypothetical proteins, Q7ZX24 of *Xenopus laevis* and Q624H0 of *Caenorhabditis briggsae* (Table 1). Due to the high degree of sequence similarity, these proteins represent homologs of HRS. Using nucleotide Blast (http://www.ncbi.nlm.nih.gov/blast/Blast.cgi), we searched the NCBI database for nucleotide sequences homologous to the *hrs* sequence from *Drosophila melanogaster*. To identify potential alternative splicing products of the *hrs* gene, we constructed a complete *Drosophila hrs* DNA sequence that includes all exons. From the identified homologous sequences, those that had been annotated as HRS/HGS were taken for further analysis. In several instances, we identified contigs that contained sequences homologous to the *Drosophila hrs* gene. To predict coding regions in these contigs, we utilized the ExPASy Genewise program (http://www.ebi.ac.uk/Wise2/index.html) using the human HRS sequence as a reference. If the contig contained a complete protein sequence, the predicted protein was taken for further analysis. Sequences encoding an identical protein were further eliminated by the ExPASy Decrease Redundancy tool (http://cn.expasy.org/tools/redundancy/). A total of 14 protein sequences were identified from various *Drosophila* species (Table 1). Interestingly, every *Drosophila* species with a known genome harbors one HRS homolog, suggesting that *hrs* is an essential gene.

### Construction of a phylogenetic tree for the HRS family of proteins

**Figure 1.**
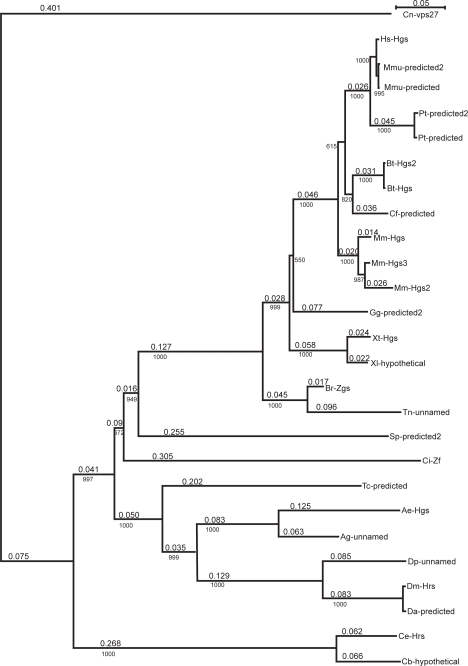
Phylogenetic tree for HRS protein family. The names of proteins are given in Table 1. Length of branch is shown above and the bootstrap value, of a branch bellow the line. The scale for branch length is given in the upper right corner. Cn-vps27 protein is an outgroup.

To understand the origin and evolution of HRS, we conducted a phylogenetic analysis of the 27 identified HRS protein sequences from 26 different taxa (Table 1) using the neighbor-joining method.^26^,^27^ The Vps27 protein sequence of *Cryptococcus neoformans* was used as an outgroup. By comparing the bootstrap support values, which correspond to the number of times a grouping occurs out of 1000 random samples from the alignment, we constructed a phylogenetic tree for the HRS family of proteins (Fig. 1). The predicted or unnamed protein sequences are likely members of the HRS family due to high bootstrap values. The order of the phylogenetic tree for the HRS clade generally coincides with the emergence of different species. The HRS family of proteins appears to have evolved from the Vps27 protein of *Saccharomyces cerevisiae*. Similar to the human and fly HRS proteins, the yeast Vps27 protein also functions in endosomal protein sorting.^28^ The HRS clade can be divided into three subgroups according to their order in evolution: worm (Q624H0 and Q17796), insect (AAPP01016184, Q960X8, Q29KX2, Q7PWQ2, Q17AG0, and XP_967857.1), and chordate (Q1RQ15, XP_001191934.1, Q4SE24, Q6PH00, Q7ZX24, Q7ZX24, XP_001191934.1, and mammalian proteins O14964, XP_001111597.1, XP_001111635, XP_511742.2, XP_001163163.1, BC111313, Q0V8S0, XP_855877.1, Q99LI8, and Q3TLL4). The predicted HRS-related sequence CBG01567 from *Caenorhabditis briggsae* is related to the HRS protein of *Caenorhabditis elegans*. Similarly, those of *Drosophila ananassae*, *simulans*, *sechellia*, *yakuba*, *pseudoobscura*, *persimilis*, *grimshawi*, and *mojavensis* are also related to each other. In addition, the unnamed sequence Q7PWQ2 of *Anopheles gambiae* and the predicted protein XP_967857.1 of *Tribolium castaneum* are closely related to the *Aedes aegypti* Hgs protein. The zinc finger protein from *Ciona intestinalis* (Q1RQ15) was the first to diverge from the rest of the chordate subgroup. HRS homologs were also found in *Strongylocentrotus purpuratus*, *Gallus gallus*, and *Canis familiaris*. Two predicted HRS isoforms were identified in *Pan troglodytes*, and three isoforms were found in *Macaca mulatta.* One of the isoforms identified in *Macaca mulatta* (Mmu-predicted2) closely resembled the human HRS protein with the exception that it contained an insertion of amino acids from position 582–672. The *Tetraodon nigroviridis* unnamed protein and the *Xenopus laevis* hypothetical protein also appeared to be members of the HRS family.

To better understand the evolutionary origin of the HRS proteins, we performed a BLAST (TBLASTN) search to identify nucleotide sequences from various taxa that were homologous to the *Drosophila* HRS protein in species whose genomic projects are represented at NCBI (http://www.ncbi.nlm.nih.gov/sites/entrez?cmd=search&db=genomeprj). Plants were represented by *Arabidopsis thaliana*; protists were represented by *Plasmodium falciparum*, *Trypanosoma cruzi*, *Dictyostelium discoideum*, *Giardia lamblia, Plasmodium falciparum*, and *Entamoeba histolytica;* fungi were represented by *Aspergillus fumigatus*, *Neurospora crassa*, *Saccharomyces cerevisiae*, *Schizosaccharomyces pombe*, and *Cryptococcus neoformans*. We used the region of highest homology and several important residues within the region to assess the degree of homology. The VHS domain-containing protein transporter NP_195002 of *Arabidopsis thaliana* showed the highest homology to the referenced *Drosophila* HRS with 48 identical residues over 140 amino acids. In contrast, the protist genomes have proteins with only short regions of homology to *Drosophila* HRS as observed in *Plasmodium falciparum* (25 identical residues versus 57 total residues), *Trypanosoma cruzi* (37 versus 75), *Dictyostelium discoideum* (29 versus 72), *Giardia lamblia* (29 versus 72), and *Entamoeba histolytica* (29 versus 72). Due to the presence of a Vps27 homolog in the fungal genomes, significantly higher homology was identified in these genomes as shown by *Aspergillus fumigatus* (87 versus 223), *Neurospora crassa* (87 versus 223), *Saccharomyces cerevisiae* (75 versus 227), *Schizosaccharomyces pombe* (85 versus 242), and *Cryptococcus neoformans* (91 versus 242). In general, our observation that fungal proteins shared a higher degree of homology with *Drosophila* HRS, compared with plants and protists, suggests that unicellular fungi are more closely related to multicellular animals and are probably closer to an animal progenitor than plants or protists (despite that plants and animals share multicellarity). If this point of view is correct, it means that the multicellularity emerged twice, once in animal lineage and once in the lineage of plants.

Multiple HRS family members were identified in *Mus musculus*, *Bos taurus*, and *Pan troglodytes*. We asked whether those proteins constitute splicing variants of the same gene or are encoded by redundant genes. Three annotated sequences Mm-Hgs2 (Q3TLL4), Mm-Hgs3 (Q3UMA3), and Mm-Hgs (Q3UMA3) were identified that encode the *Mus musculus* HRS protein. The Mm-Hgs2 and Mm-Hgs3 sequences differ from each other by a 30-residue fragment at the C-terminal region of HRS. It is highly probable that these two sequences represent alternatively spliced mRNA isoforms encoded by the murine HRS gene. Mm-Hgs differs from Mm-Hgs2 and Mm-Hgs3 by eight amino acids dispersed throughout the protein, indicating that two HRS genes may exist in the murine genome. The *Bos taurus* HRS protein is represented by two sequences, Bt-Hgs and Bt-Hgs, which differ from each other by one amino acid (at position 359 of alignment) and a deletion of one amino acid (position 450 of alignment). Most probably, these differences are not due to the existence of two different genes but rather result from reading errors. The *Pan troglodytes* HRS sequences, Pt-predicted and Pt-predicted2, differ from each other only by a contiguous amino acid sequence (positions 594–675 of alignment), suggesting that these sequences may be alternative splicing isoforms. Similarly, the *Macaca mulatta* HRS sequences [Mmu-predicted, Mmu-predicted2, and Mmu-predicted3 (identical to a human protein sequence)] may represent alternative splicing isoforms of a single gene as they differ from each other only by a contiguous amino acid sequence. Thus, our phylogenetic study revealed redundant HRS genes only in the *Mus musculus* species, possibly reflecting specific properties of chromosome evolution rather than evolutionary pressure on the HRS gene.

### Evolution of the functionally important residues in HRS

**Figure 2.**
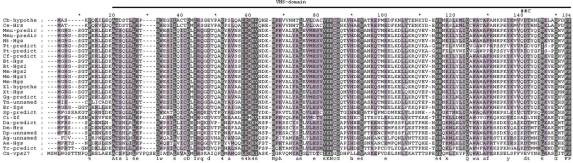
Alignment of the VHS-domain. The depth of three-level shadow is directly proportional to the degree of evolutionary conservation. The bar above the alignment shows the position of VHS domain. *—Intermediate enumerator. ##C—an alternative splicing site of Drosophila gene; the symbols bellow the alignment show the degree conservation of alignment positions (abbreviated as in GeneDoc program).

Although the initial identification of proteins via sequence similarities does not yield a clear indication of their respective functions, analysis of specific conserved regions and residues may provide important information regarding their putative functional characteristics. The known domains of HRS protein are denoted in our alignment according to its location in human HRS (Supplement 1). The HRS protein consists of five protein domains: the VHS domain, the FYVE domain, the UIM domain, the NF2-binding domain, and the clathrin-binding domain. Commonly found in Vps27, HRS, and STAM proteins^9^ and known to function in cargo recognition in the trans-Golgi network (TGN), the VHS domain is likely involved in general membrane targeting/cargo recognition during vesicular trafficking, consists of eight helices arranged in a superhelix, and interacts with the FYVE domain of human and *Drosophila* HRS.^29^,^30^ This domain spans 21–154 residues of the alignment and has 11 phylogenetically conserved positions (Fig. 2). Nine non-conserved residues separate the VHS domain from the FYVE domain.

**Figure 3.**
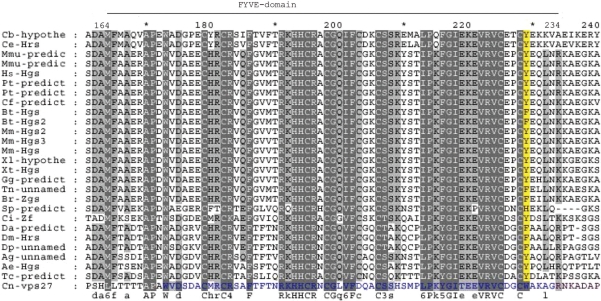
FYVE-domain alignment. The abbreviations as in Figure 1. The FYVE domain of *Cryptococcus neoformans* is shown by blue letters; phosphorylation site, with yellow.

The FYVE zinc-finger—like domain was shown to be responsible for the ability of HRS to bind to PtdIns 3-phosphate (PtdIns(3)P) in the intracellular membranes.^11^ It has been shown that EEA1 (Early Endosome Associated protein 1) protein requires its FYVE finger for endosomal localization.^31^ Since the early endosomal localization of *Drosophila* HRS protein is already well-established,^6^ the FYVE domain appears to be most important for this intracellular localization. In our alignment, FYVE is located at positions 166–234, contains 23 conserved residues, and appears to be the most conserved domain of this protein family. Since the location of FYVE is also known for the *Saccaromyces cerevisiae* protein,^31^ we aligned the *Saccaromyces cerevisiae* and *Cryptococcus neoformans* Vps27 sequences and inferred the location of the FYVE domain in *Cryptococcus neoformans* (blue letters in the *Cryptococcus neoformans* sequence in Fig. 3); the data displayed a good fit.

**Figure 4.**
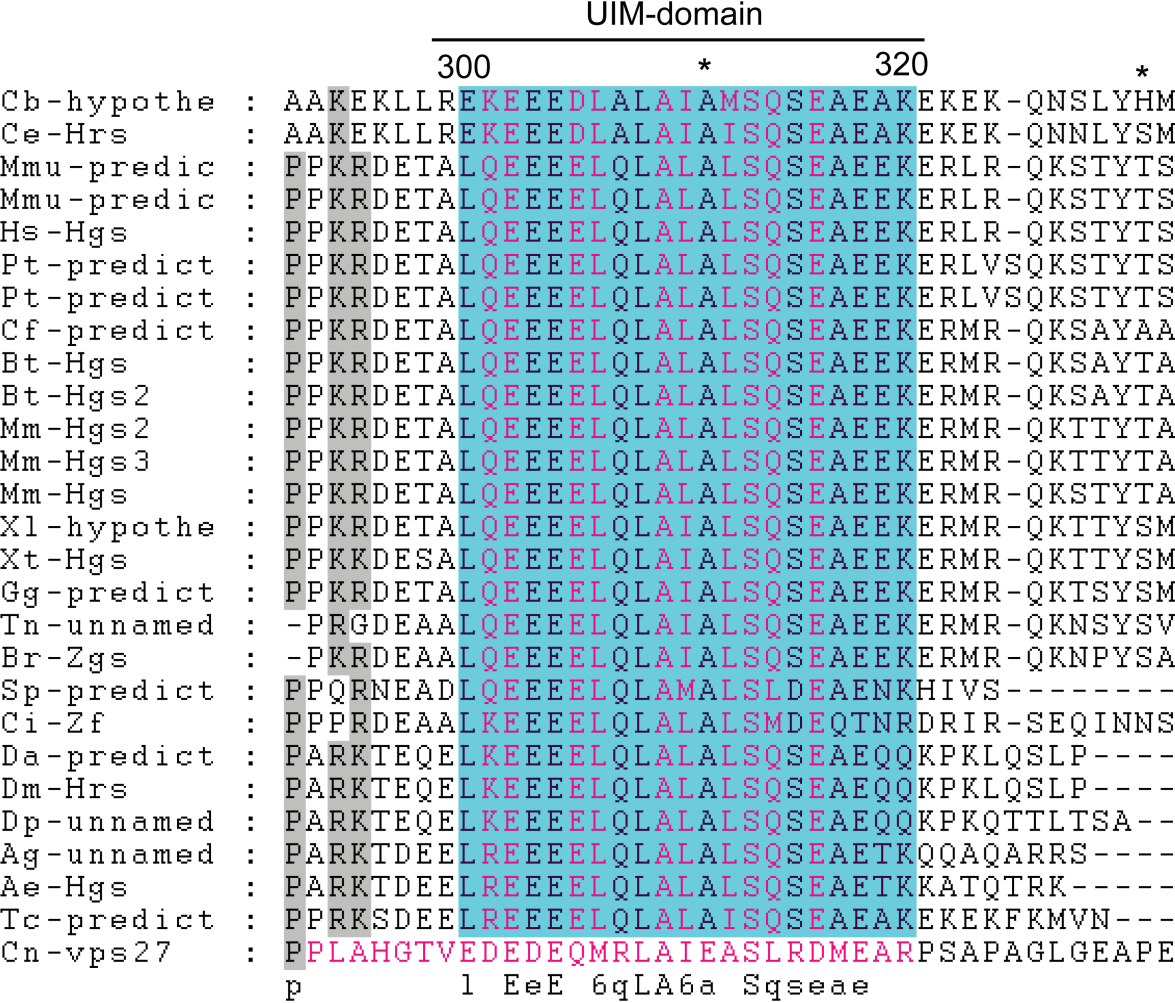
UIM-domain alignment. Abbreviations as in Figure 1. Amino-acids belonging to UIM domain are blue shadowed. Ubiquitin interacting aminoacids are denoted by orange letters. UIM domain of Vps27 protein differ from UIM domains of HRS protein and is denoted by red letters.

Ubiquitination of membrane proteins is a signal for internalization and endosomal sorting and constitutes an important mechanism for down-regulation of activated cell surface receptors.^32^ The HRS protein contains a UIM domain that is commonly found in endocytic proteins. Point mutations in the UIM domain result in defective sorting of ubiquitinated proteins to the vacuolar lumen, while deletions of the UIM domain do not alter the recycling of Golgi proteins and the generation of luminal membranes, suggesting that Vps27 has multiple functions in the endosome.^7^,^28^ Alignment of the HRS proteins with the Vps27 protein indicated that UIM occupies positions 301–321 of Vps27 and contains five residues that are conserved from yeast to humans. The alignment suggests that these conserved residues function as direct intermolecular contacts in the interaction between ubiquitin and the Vps27 UIM (the intermolecular contacts are denoted with yellow letters in the UIM domain in Fig. 4 according to Swanson et al).^33^ Notably, the Vps27 protein has an additional UIM domain (both UIM domains are marked with red letters in the Vps27 sequence). This domain binds one ubiquitin molecule, while the UIM domain of HRS binds two.^34^ If the UIM domain in HRS simply acquired the function of two mono-ubiquitin sites of Vps27, the conservation of the first UIM site in the HRS sequence (denoted Box1 in our alignment) is unusual. Most probably, this conserved, likely unnecessary, UIM site has some unknown function that has not yet been described.

**Figure 5.**
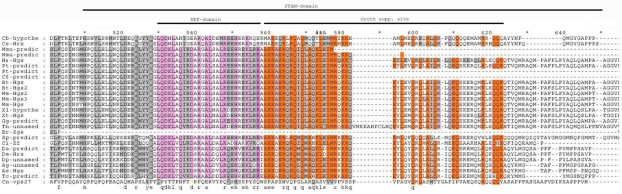
STAM domain alignment. Abbreviations as in Figure 1. Conserved residues of NF2 interacting domain denoted by pink color and the conserved residues of growth suppression domain by orange color. The phosphoserine (539 position of alignment) in *Cryptococcus neoformans* sequence denoted with yellow letter.

Similar to HRS, the STAM (signal-transducing adaptor molecule) protein was identified as a tyrosine phosphorylated protein in cells stimulated with growth factors or cytokines.^1^,^2^,^35^–^38^ Mammals have two STAM genes, encoding homologous STAM1 and STAM2 proteins. Based on the alignment of HRS protein family members, the STAM interacting domain of the HRS protein occupies positions 511–646.^2^ Residues within this region are conserved in multicellular organisms (Fig. 5); however, no conserved residues between yeast and animals were detected in our alignment. To determine whether a STAM homolog exists in yeast, a BLAST search was performed using the sequence for the human STAM1 protein. The resulting protein, YHL002W of *Saccharomyces cerevisiae*, exhibits homology to mammalian STAM proteins, and the conserved residues are dispersed throughout the protein sequence, showing that the homology is not restricted to a particular domain. The STAM protein is a subunit of the endosomal Vps27–Hse1 complex, which is required for the sorting of ubiquitinated membrane proteins into intraluminal vesicles prior to vacuolar degradation as well as for recycling of Golgi proteins and formation of luminal membranes. Thus, it is highly probable that the STAM protein of multicellular animals evolved from a vesicular trafficking protein similar to YHL002W and concurrently acquired a new function in signal transduction. The absence of conserved residues from yeast to animals can be explained by the numerous amino acid changes needed to acquire a new function.

Consistent with Sun et al,^21^ the Merlin-interacting domain of HRS is situated at positions 530–556 of our alignment and contains a stretch of 10 amino acid residues that are conserved among multicellular animals, suggesting that this region may be functionally important. Interestingly, the serine at residue 539 of the alignment in the *Cryptococcus neoformans* sequence was substituted with aspartic acid in all other sequences, except in worms. Since aspartic acid is thought to mimic a constitutively phosphorylated position,^39^ it is hypothesized that a multicellular progenitor acquired an aspartic acid at this position to use only a “phosphorylated” form of the protein rather than either the phosphorylated or nonphosphorylated form. The presence of glutamic acid at this position in worms may represent a unique amino acid change in this particular lineage. Previously, we showed that the Merlin protein first appeared in multicellular animals, namely worms.^24^ Our analysis clearly demonstrates that the transition from unicellular to multicellular organisms was accompanied not only by changes in the phosphorylation state of the HRS protein, but also by the appearance of a Merlin-binding domain. These results suggest that the protein “machinery” regulating proliferation, including Merlin and HRS, was established during the transition to multicellarity and evolved from precursor proteins of unicellular eukaryotes.

Over-expression of truncated HRS proteins in RT4 rat schwannoma cells leads to growth suppression. The HRS domain responsible for growth suppression was mapped to positions 498–551 of the human protein^21^ and to positions 559–622 of our alignment. The conserved amino acids among various HRS proteins are concentrated between positions 559 and 579, suggesting that this region is crucial for growth suppression. After position 579, the homology decreases and is frequently interrupted by amino acid deletions, indicating that the residues after position 579 are less important for growth suppression.

**Figure 6.**
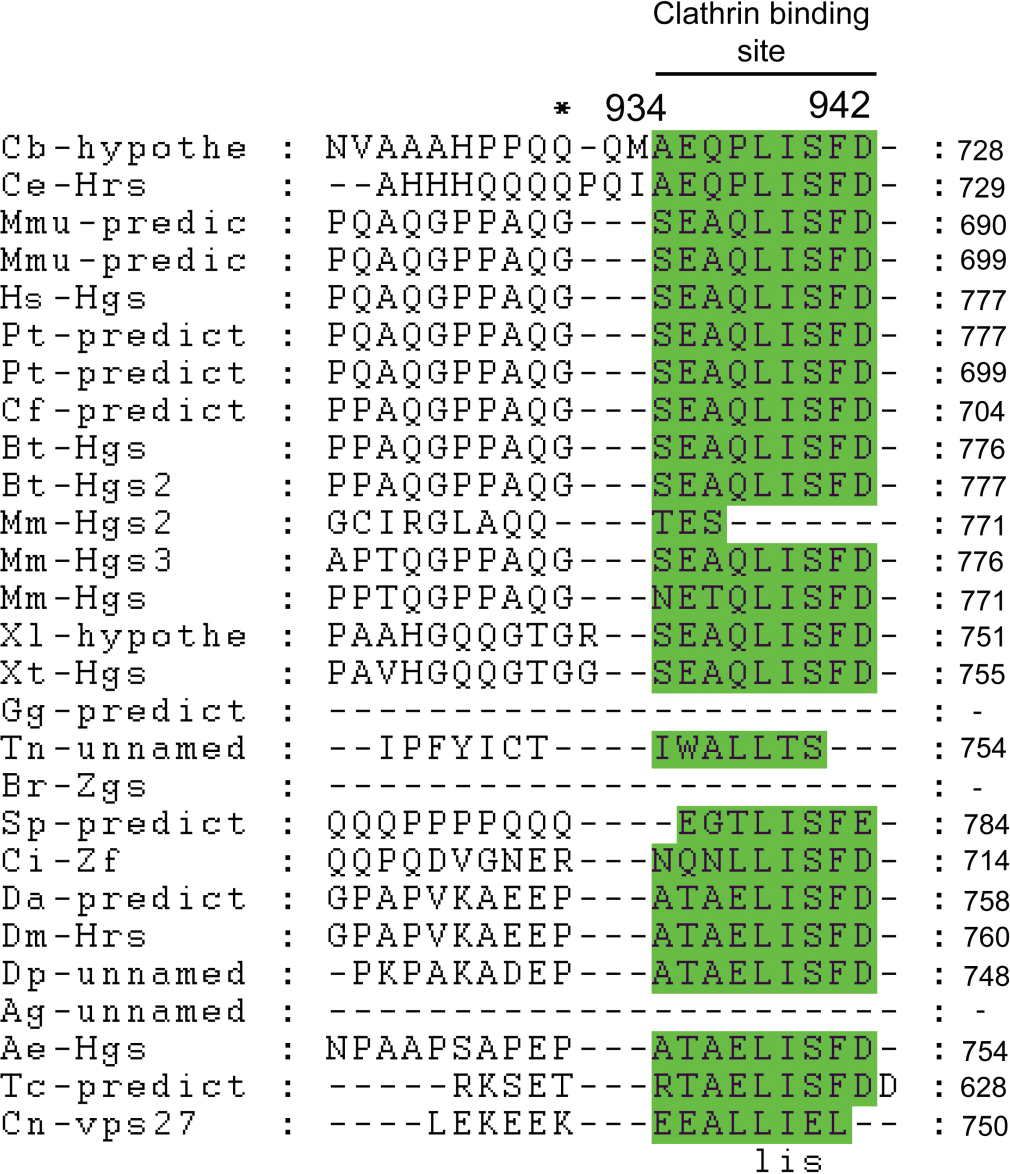
Clathrin binding domain alignment. Abbreviations as at Figure 1.

Colocalization of HRS and clathrin suggests a functional importance for the clathrin-binding site of HRS,^8^,^40^ which is located at positions 934–944 of our alignment and contains three conserved positions (Fig. 6). The absence of the clathrin-binding site in the *Brachydanio rerio* (Q6PH00) and *Anopheles gambiae* (Q7PWQ2) proteins may be due to sequence determination or ORF prediction at the C-terminal ends (these data were marked in the ExPASy as preliminary data) rather than the absence of this conserved domain from these proteins.

As evident from the studies of Bean et al^41^ and Raiborg et al,^15^ HRS can bind to Eps15, which is an endocytosis regulator, and the sorting nexin 1 (Snx1) protein, which interacts with the domain of EGFR that confers lysosomal targeting. The HRS domains responsible for binding to Eps15 and Snx1 have been located between the FYVE and STAM-binding domains.^15^ Sequence alignment revealed two conserved regions (Box1 and Box3 in Supplement 1) that may be important for binding to the Eps15 and Snx1 proteins.

The phosphorylation patterns of the human HRS and *Saccaromyces cerevisiae* Vps27 proteins have been characterized by mass spectrometry.^42^–^44^ Using the alignment of the *Saccaromyces cerevisiae* and *Cryptococcus neoformans* proteins, we compared the positions of phosphorylated residues in the animal and yeast HRS proteins. The serine residue found at position 157 of the *Saccaromyces cerevisiae* Vps27 corresponds to a valine at position 159 of the *Cryptococcus neoformans* protein (position 160 of the alignment). Also, a residue that can be phosphorylated was not found at the equivalent position of the animal HRS proteins. The tyrosine residue at position 229 of the alignment, corresponding to residue 216 in the human HRS protein, is substituted by a phenylalanine in the *Bos taurus* (Q2NKZ6 and Q0V8S0), *Brachydanio rerio* (Q6PH00), *Ciona intestinalis* (Q1RQ15), *Drosophila melanogaster* (Q960X8) and *pseudoobscura* (Q29KX2), and *Anopheles gambiae* (Q7PWQ2) sequences and by a tryptophan in the *Cryptococcus neoformans* (Q5KGG4) sequence. These results suggest that phosphorylation at this position is not a characteristic feature of the HRS protein family. However, the tyrosine residue at position 356 of the alignment (position 308 of the human protein) is conserved in vertebrates but absent in *Strongylocentrotus purpuratus, Ciona intestinalis, Schistosoma japonicum*, and in insects and worms. Located within Box2, the tyrosine-329 residue of human HRS (position 377 of the alignment) is highly conserved throughout the entire protein family, suggesting that Box2 may be functionally important for phosphorylation. This evidence is consistent with the phosphorylation sites identified in the yeast protein in response to pheromone treatment.^44^ In addition, although the tyrosine-334 residue of the human protein (position 334 of the alignment) is conserved across many taxa, the *Ciona intestinalis* protein contains an alanine, and the yeast Vps27 protein has a tryptophan at this position. Since alanine and tryptophan cannot be phosphorylated, the tyrosine residue at this position may be a phosphorylation site but only for some taxa. Similarly, the serine-495 residue of the yeast Vps27 protein, which corresponds to serine-499 in the *Cryptococcus neoformans* protein and is located at position 539 of the alignment (yellow letter in the *Cryptococcus neoformans* sequence), is not conserved, as proteins from other species have an amino acid at this position that cannot be phosphorylated.

### Exon–intron structural evolution and alternative splicing of the HRS gene

We used the genome contigs from the GenBank database and protein sequences predicted from EST or complete mRNA sequences (Table 2) to determine the exon–intron structure of the *HRS* genes from various species. The GeneWise2 program (http://www.ebi.ac.uk/Tools/Wise2/index.html) was used to search for the amino-acid sequences encoded by the different exons (Supplement 2). The mammalian *HRS* gene contains 22 exons, whereas the *Drosophila hrs* gene consists of only five exons. This observation coincides with our previous finding that mammalian Merlin genes contain more introns than those of insects and that introns of mammalian Merlin genes are longer than those of the insect Merlin genes.^24^

In Table 2, human HRS is represented by two alternative splicing isoforms (AF260566 and O14964). The short isoform AF260566 is spliced between residues 517 and 605 of the O14964 sequence (positions 573 and 681 of our alignment), and the long isoform O14964 is spliced between residues 524, 525 and 569, 570. Alignment of residues 517–605 (residues 517 and 605 are designated as ##A and ##B (575 and 681 positions of alignment) in Supplement 1, respectively) with the other sequences shows that this alternative splicing results in deletion of the conserved residues at the C-terminal end of the growth suppression domain and, possibly, the STAM binding domain. A similar deletion of these conserved residues occurs in the *Pan troglodytes* isoform 1 (XP_001163163.1) and *Macaca mulatta* isoforms 1 and 2 (XP_001111597.1 and XP_001111635.1). These results suggest that such a change may have some functional significance. Analysis of the region between positions ##A and ##B shows that it is variable among various HRS proteins and is partially absent in the insect proteins. Previously, Scoles et al^20^ used the yeast two-hybrid system to demonstrate that the long isoform of HRS binds to Merlin with a higher affinity than the short isoform and that residues 363–469 of Merlin are responsible for the differential binding to the long and short isoforms of HRS. Thus, the ability of different HRS isoforms to bind Merlin with varying affinities may be important in specific evolutionary and/or developmental contexts.

The *Drosophila* HRS long and short isoforms (Q960X8 and NP_722831.1) are generated by alternative splicing (shown in Supplement 2). The short isoform appears if the small coding region located within intron 3 of the gene is added to this protein. The positions of the protein family alignment corresponding to this alternative splicing site are denoted as **##C** (140 position of alignment) in Supplement 1 (also see Supplement 2). As a result of this splicing, the VHS domain is almost completely absent. Using ExPASy, we identified four human VHS domain-containing proteins that differ from the VHS domain of HRS: (1) GGA2 (Q9UJY4), encoding an ADP-ribosylation factor—binding protein, (2) GGA1 (Q86YA9), encoding the Golgi-associated, gamma-adaptin, (3) the “ear-containing” ARF-binding protein 1 (Q8NCS6), and (4) GGA3 (Q9NZ52), also encoding an ADP-ribosylation factor–binding protein. While the roles of GGA1 and ARF binding protein 1 are unknown, the GGA2 and GGA3 proteins primarily function in protein sorting and trafficking between the trans-Golgi network and endosomes. Since HRS and the GGA2 and GGA3 proteins are involved in protein sorting and endocytosis, the absence of a VHS domain in the *Drosophila* HRS short isoform suggests that this isoform may not have the ability to bind to membranes.

## Conclusions

Our results demonstrate a monophyletic origin of the HRS protein in the early Metazoa, similar to our previous finding for the *NF2* tumor suppressor protein Merlin.^24^ The HRS protein domains involved in intracellular vesicular trafficking include the VHS, FYVE, UIM, and clathrin-binding domains, which are conserved from yeast to mammals. Tyrosine-377, a potential phosphorylation site, is conserved throughout the entire protein family, and phosphorylation at this site is likely to be connected to HRS function in vesicular trafficking. Using the STAM-binding, Merlin-binding, and growth suppression domains of HRS that have been mapped,^2^,^21^ we showed that these domains and a number of potential phosphorylation sites are absent in unicellular eukaryotes and that they first appear in the Metazoan lineage. Analysis of the exon/intron structure of the HRS genes shows the existence of the splicing variants lacking the VHS domain. Thus, it appears that the function of this domain can depend on the evolutionary and/or developmental context. The existence of HRS splicing variants lacking almost the entire STAM-binding and growth suppression domains suggest that some HRS proteins may not have these functions. Further experimental investigation is warranted to better understand the function and evolution of the HRS protein and its interaction with Merlin.

## Figures and Tables

**Table 1.** Protein sequences showing homology to human HRS.

**Numerical identifier for this article**	**Short name for this article**	**Species and protein family names (if a protein family name was given in database)**	**Accession number**	**Identity (the sequence is identical with)**
			Accession number in Expassy	
1	Dm-Hrs	*Drosophila melanogaster* Hrs	Q960X8	
2	Dp-unnamed	*Drosophila pseudoobscura*	Q29KX2	
3	Ag-unnamed	*Anopheles gambiae*	Q7PWQ2	
4	Ae-Hgs	*Aedes aegypti* Hgs	Q17AG0	
5	Xl-hypothetical	*Xenopus laevis* Hypothetical protein	Q7ZX24	
6	Bt-Hgs	*Bos taurus* Hgs	BC111313	
7	Bt-Hgs2	*Bos taurus* Hgs	Q0V8S0	
9	Mm-Hgs2	*Mus musculus* Hgs HGF-regulated tyrosine kinase substrate	Q3TLL4	8 Mm-Hgs (Q3UMA3)
12	Tn-unnamed	*Tetraodon nigroviridis*	Q4SE24	
13	Mm-Hgs3	*Mus musculus* Hgs HGF-regulated tyrosine kinase substrate	Q99LI8	10 Hgs Rat (Q9JJ50)
14	Rn-Hgs	*Rattus norvegicus* Hgs Hepatocyte growth factor-regulated tyrosine kinase substrate. SNAP-25-interacting protein Hrs-2	Q9JJ50	
15	Xt-Hgs	*Xenopus tropicalis* Hgs Hepatocyte growth factor-regulated tyrosine kinase substrate	Q28CS1	
16	Ci-Zf	*Ciona intestinalis* Zinc finger protein	Q1RQ15	
17	Br-Zgc	*Brachydanio rerio* Zgc:63492	Q6PH00	
18	Cb-hypothetical	*Caenorhabditis briggsae* Hypothetical protein CBG01567	Q624H0	
19	Ce-Hrs	*Caenorhabditis elegans* Hepatocyte growth factor-regulated tk substrate (Hrs) family protein 1	Q17796	
20	Sj-SJCH	*Schistosoma japonicum* SJCHGC04426 protein	Q5C033	
21	Cn-vps27	*Cryptococcus neoformans* Vacuolar protein sorting-associated protein vps27, putative	Q5KGG4	
41	Hs-Hgs	*Homo sapiens*	O14964 Accession number in NCBI	*Macaca mulatta* (XP_001111673.1)^+^
34	Pt-predicted^+^	*Pan troglodytes*	XP_511742.2	23 *Pan troglodytes* (XP_540486.2)^+^
36	Pt-predicted2^+^	*Pan troglodytes*	XP_001163163.1	25 *Pan troglodytes* (XM_001163163)^+^
38	Rn-predicted^+^	*Rattus norvegicus*	XP_340795.2	26 *Rattus norvegicus* (XM_00107140527)^+^ (XM_340794)^+^
39	Mmu-predicted^+^	*Macaca mulatta*	XP_001111597.1	28 *Macaca mulatta* (XP_001111597.1)^+^
40	Mmu-predicted2		XP_001111635	29_*Macaca mulatta* (XP_001111635)^+^
	*Macaca mulatta*		XP_967857.1	
42	Tc-predicted^+^	*Tribolium castaneum*	XP_426233.1	
43	Gg-predicted2^+^	*Gallus gallus*		
44	Cf-predicted^+^	*Canis familiaris*	XP_855877.1	22 *Canis familiaris*
46	Sp-predicted^+^	*Strongylocentrotus purpuratus*	XP_790636.2	45_*Strongylocentrotus purpuratus* (XP_001184136.1)^+^
47	Sp-prdicted2^+^	*Strongylocentrotus purpuratus*	XP_001191934.1	48_*Strongylocentrotus purpuratus* (XP_783582.2)^+^
50	Ds-predicted*	*Drosophila simulans* strain MD106TS	AASS01027272	
53	Ds-predicted2*	*Drosophila simulans* strain MD199S	AAST01008328	
54	Ds-predicted3*	*Drosophila simulans* strain SIM6	AASW01048297	
59		*Drosophila virilis* strain TSC#15010-1051.87	AANI01016223	
63	Da-predicted*	*Drosophila ananassae* strain TSC#14024-0371.13	AAPP01016184	49 *Drosophila simulans* (AAGH01016410)*
				51 *Drosophila sechellia (AAKO01001385*)*
				52 *Drosophila yakuba* (AAEU02000364)*
				55 *Drosophila pseudoobscura* (AADE01000840)*
				56 *Drosophila pseudoobscura* (AAFS01000141)*
				57 *Drosophila persimilis* (AAIZ01003953)*
				58 *Drosophila grimshawi* (AAPT01021698)*
				60 *Drosophila mojavensis* (AAPU01010653)*
				61 *Drosophila simulans* (AASS01027271)*
				62 *Drosophila simulans* (AAST01008327)*

+ **Notes:** Proteins predicted by automated computational analysis, using gene prediction method: GNOMON, supported by mRNA and EST evidence.

* Proteins predicted by a Genewise program (in this article) in the genomic contigs.

**Table 2.** Protein sequences supported by mRNA evidence (EST or mRNA sequencing data).

**Accession number**	**Species and protein name or symbol**
AF260566	*Homo sapiens* HRS isoform 2 (HRS) mRNA
O14964	*Homo sapiens*—Hgs
XP_511742.2	*Pan troglodytes* predicted HGS isoform 2
XP_001163163.1	*Pan troglodytes* predicted HGS isoform 1
XP_001111597.1	*Macaca mulatta* predicted HGS isoform 1
XP_001111673.1	*Macaca mulatta* predicted HGS isoform 3
XP_001111635.1	*Macaca mulatta* predicted HGS isoform 2
BC111313	*Bos taurus* HGS mRNA
Q0V8S0	*Bos taurus*-Hgs2
Q99LI8	*Mus musculus*-Hgs3
Q960X8	*Drosophila melanogaster*-HRS
NP_722831.1	*Drosophila melanogaster* isoform A
